# Effective Signal Extraction Algorithm for Cerebral Blood Oxygen Based on Dual Detectors

**DOI:** 10.3390/s24061820

**Published:** 2024-03-12

**Authors:** Zhiming Xing, Zihao Jin, Shuqi Fang, Xiumin Gao

**Affiliations:** School of Optical-Electrical and Computer Engineering, University of Shanghai for Science and Technology, Shanghai 200020, China233350728@st.usst.edu.cn (S.F.)

**Keywords:** fNIRS, GA-VMD, dual detectors, information extraction, hemoglobin

## Abstract

Functional near-infrared spectroscopy (fNIRS) can dynamically respond to the relevant state of brain activity based on the hemodynamic information of brain tissue. The cerebral cortex and gray matter are the main regions reflecting brain activity. As they are far from the scalp surface, the accuracy of brain activity detection will be significantly affected by a series of physiological activities. In this paper, an effective algorithm for extracting brain activity information is designed based on the measurement method of dual detectors so as to obtain real brain activity information. The principle of this algorithm is to take the measurement results of short-distance channels as reference signals to eliminate the physiological interference information in the measurement results of long-distance channels. In this paper, the performance of the proposed method is tested using both simulated and measured signals and compared with the extraction results of EEMD-RLS, RLS and fast-ICA, and their extraction effects are quantified by correlation coefficient (R), root-mean-square error (RMSE), and mean absolute error (MAE). The test results show that even under low SNR conditions, the proposed method can still effectively suppress physiological interference and improve the detection accuracy of brain activity signals.

## 1. Introduction

Functional near-infrared spectroscopy (fNIRS) is a new noninvasive imaging technique to analyze the state of the human body by detecting changes in blood parameters [1,2]. Near-infrared detection technology has been widely used in many fields [3]. fNIRS is now widely used to obtain hemodynamic information such as on oxygenated hemoglobin (Oxy-Hb) and deoxyhemoglobin (Deoxy-Hb). Compared with other brain function detection techniques [4] such as electroencephalogram (EEG), magnetoencephalography (MEG), positron emission tomography (PET), and functional magnetic resonance imaging (fMRI) fNIRS technology has the advantages of simple structure, low cost, easy-to-realize miniaturization, and noninvasiveness and non-stimulation [5], and therefore has obvious advantages in the detection of signals from various physiological activities. Currently, fNIRS is widely used in clinical practice; for example, fNIRS is used to help the study of processes related to neurological and psychiatric disorders, such as Alzheimer’s disease, Parkinson’s disease, epilepsy, schizophrenia, and anxiety disorders [6]. Recently, fNIRS has also been considered for brain computer interfaces [7,8].

Due to neurovascular coupling mechanisms, changes in Oxy-Hb and Deoxy-Hb concentrations occur in response to specific stimuli or cognitive tasks. fNIRS monitors these physiological parameters by measuring changes in light intensity as it passes through the brain tissue. This is achieved using a beam of red and near-infrared light. The estimated hemodynamic response can be interpreted as an indirect measure of neural activity in the studied brain region [9].

However, due to the cerebral cortex and gray matter portion of the brain being far away from the surface layer of the scalp, the measured signals are inevitably affected by extracerebral and self-physiological components when using fNIRS to detect brain functions. In actual measurements, the measurement accuracy of fNIRS can be affected by a variety of factors, such as insufficient photon penetration depth [10], improper arrangement of the relative positions of the light source and the detector [11], and physiological interference of the human body [12]. These physiological disturbances mainly include heartbeats, breathing, low-frequency oscillations, Mayer waves, and a number of other disorganized interference signals [13,14]. These interferences are distributed in the scalp, skull, and internal brain tissues, so it is inevitable to be affected by these interferences when using fNIRS to measure blood oxygenation levels [15]. Moreover, it has been shown that there are individual differences in the correlation between the fNIRS signal and scalp blood flow or mean blood pressure [16], and systemic changes affecting the extracranial signal may also lead to false positives in the fNIRS signal [17]. Therefore, the question of how to eliminate the influence of these physiological interferences on the measurement results of fNIRS is particularly important.

At present, the commonly used methods to eliminate physiological interference include band pass filters [18,19], independent component analysis (ICA) [20], adaptive filtering (RLS) [21], and generalized linear modeling (GLM) [22]. Saager et al.‘s study on brain tissue showed [23] that about 10% of the subjects’ brains showed heterogeneity, at which time the traditional brain signal analysis methods were no longer applicable. In order to solve this problem, some studies have proposed using the generated signal as a reference signal and combining the collected signal to estimate the real signal parameters [24]. This method provides a feasible idea for extracting cerebral blood oxygen signals, but it is not universally applicable due to the differences between individuals and the need to accurately model the reference signal according to the type and nature of interference.

It has been shown that the detection range of the detector to the brain tissue is numerically related to the distance between the light source and the detector [25,26]. The greater the distance between the light source and the detector, the easier it is to detect photons scattered from deep brain tissue. Recently, the dual-channel measurement method has become an effective method for eliminating physiological interference [27]. The common practice is to multiply the signal from the short-range measurement channel by a weighting factor and subtract this value from the long-range channel. These methods are often used in conjunction with methods such as static linear regression. However, if there is a correlation between the deep and shallow signals, the deep signal may be over-subtracted by the regression of the signal from the short-range channel. In addition, this method increases computational complexity. Katerina Barnova et al. proposed a noninvasive fetal ECG extraction algorithm based on Ensemble Empirical Mode Decomposition (EEMD) [28], which utilizes a combination of ICA, RLS, and EEMD to extract fetal ECG signals from abdominal recordings. In Reference [29], a method based on Empirical Mode Decomposition (EMD) for fetal ECG extraction was proposed. Modal aliasing often occurs in the decomposition results of EMD, which also has an impact on the final measurement results.

Aiming at the problems with the previous extraction methods, we designed a new method which can be used to extract the brain activity information related to external stimuli from the cerebral blood oxygenation signals measured with a dual-channel fNIRS device (including a short-range measurement channel and a long-range measurement channel). This method does not require setting up the reference signal in advance and updating the weights of each component, and the method has a certain degree of performance enhancement compared with previous methods, both in the computational process and in the extraction results. First, the method is used to decompose the acquired signals of long- and short-distance channels modally, obtain several different frequency components, and select the high-frequency and low-frequency components by combining the signal characteristics and frequency spectrum. Next, the high-frequency and low-frequency components are processed separately with the proposed method, and the interference information contained in the long-distance channel can be removed so as to obtain the real brain activity information. In order to evaluate the performance of the proposed method, this paper also compares the extraction results with those of EEMD-RLS [30], fast-ICA [31], and RLS [32]. In this paper, the correlation coefficient (R), root-mean-square error (RMSE), and mean absolute error (MAE) are used to evaluate the extraction results of different methods. The results show that even under low signal-to-noise ratio conditions, the proposed method still outperforms the compared methods in both waveform shape restoration and amplitude recovery.

## 2. Theory and Methods

### 2.1. Variational Mode Decomposition

Variational mode decomposition (VMD) is an adaptive and completely non-recursive mode variation and signal processing method [33]. In signal processing, this method is used to determine the frequency center and bandwidth of each component by iteratively searching for the optimal solution of the variational model during the process of obtaining the decomposition components, so that the frequency domain of the signal can be self-adaptive and the effective separation of each component can be realized. The process of VMD decomposition mainly involves the Wiener filter, the Hilbert transform, and frequency mixing. By using the Wiener filter and Hilbert transform, the decomposition problem of the original signal is converted into a variational problem of solving K modes. In order to minimize the sum of estimated bandwidths of each mode, a constrained variational model is established, whose expression is as follows:

(1) $$\begin{array}{l} \underset{\left\{u\},\{\omega \right\}}{\min}\left\{{\sum_{i=1}^{k}\left\|\partial_{t}\left\{\left[\delta (t)+\frac{j}{\pi t}\right]\times u_{i}(t)\right\}e^{-j\omega_{i}t}\right\|}_{2}^{2}\right\}\\ s.t.\sum_{i=1}^{k}u_{k}=f(t) \end{array}$$

where $\left\{u\}=\{u_{1},\cdots ,u_{k}\right\}$ denotes the *k* Intrinsic Mode Function (IMF) components, $\left\{\omega \}=\{\omega_{1},\cdots ,\omega_{k}\right\}$ denotes the central frequency set of the *k* components, δ indicates a Dirac distribution, $\left[\delta (t)+\frac{j}{\pi t}\right]\times u_{i}(t)$ represents the unilateral spectrum of the analytic signal obtained by the Hilbert transform of $u_{i}(t)$, ${\left\|\partial_{t}\left\{\left[\delta (t)+\frac{j}{\pi t}\right]\times u_{i}(t)\right\}e^{-j\omega_{i}t}\right\|}_{2}^{2}$ denotes the bandwidth of each IMF estimated by calculating the square of the modulated signal gradient norm L2, f(t) is the original signal, and t is an arbitrary moment. The constrained problem in Equation (1) is converted into an unconstrained variational problem by introducing a quadratic penalty factor α and the Lagrange multiplier operator λ(t). The extended augmented Lagrange expression is as follows:

(2) $$\begin{array}{ll} L\left\{\left\{u\right\},\left\{\omega \right\},\lambda (t)\right\} & =\alpha {\sum_{i=1}^{k}\left\|\partial_{t}\left\{\left[\delta (t)+\frac{j}{\pi t}\right]\times u_{i}(t)\right\}e^{-j\omega_{i}t}\right\|}_{2}^{2}+{\left\|f(t)-\sum_{i=1}^{k}u_{i}(t)\right\|}_{2}^{2}\\ & +\langle \lambda (t),f(t)-\sum_{i=1}^{k}u_{i}(t)\rangle \end{array}$$

By continuously updating $u_{k}^{\left(n+1\right)}$, $\omega_{k}^{\left(n+1\right)}$, and $\lambda_{k}^{\left(n+1\right)}$ for each IMF, the saddle point of Equation (2) is simultaneously found and taken as the solution of Equation (1). The expressions required to alternately find the optimal $u_{k}$, $\omega_{k}$, and $\lambda_{k}$ values are as follows:

(3) $${\hat{u}}_{k}^{\left(n+1\right)}(\omega )=\frac{\hat{f}(\omega )-\sum_{i=1}^{k-1}\left[{\hat{u}}_{i}(\omega )+\hat{\lambda }(\omega )/2\right]}{1+2\alpha {\left(\omega -\omega_{k}\right)}^{2}}$$

(4) $$\omega_{k}^{\left(n+1\right)}=\frac{\int_{0}^{\infty }\omega {\left|{\hat{u}}_{k}(\omega )\right|}^{2}d\omega }{\int_{0}^{\infty }{\left|{\hat{u}}_{k}(\omega )\right|}^{2}d\omega }$$

(5) $${\hat{\lambda }}^{\left(n+1\right)}(\omega )={\hat{\lambda }}^{\left(n\right)}(\omega )+\tau \left[\hat{f}(\omega )-\sum_{i=1}^{k}{\hat{u}}_{i}^{n+1}(\omega )\right]$$

where ${\hat{u}}_{k}^{\left(n+1\right)}(\omega )$, $\hat{f}(\omega )$, and $\hat{\lambda }(\omega )$ denote the corresponding Fourier transforms of $u_{k}^{\left(n+1\right)}(t)$, f(t), and λ(t), respectively, *n* denotes the number of iterations, τ denotes the step size, and the central frequency of each component $u_{i}$ is estimated using the center of gravity of the power spectrum during the iterative calculation. The iteration termination condition is shown in Equation (6), and when the iteration error is less than the threshold ε, the iteration is stopped and the output modal component is $\left\{u_{k}\right\}$ and the estimated modal center frequency is $\left\{\omega_{k}\right\}$.

(6) $$\sum_{k=1}^{K}\frac{{\left\|{\hat{u}}_{k}^{\left(n+1\right)}(\omega )-{\hat{u}}_{k}^{\left(n\right)}(\omega )\right\|}_{2}^{2}}{{\left\|{\hat{u}}_{k}^{\left(n\right)}(\omega )\right\|}_{2}^{2}}<\varepsilon$$

### 2.2. GA-VMD

According to the above analysis, the initial parameters *K*, α, τ, and ε of VMD decomposition must be defined in advance. The number of decompositions *K* and the quadratic penalty term α have a significant impact on the VMD decomposition results, so the parameters need to be optimized by using an optimization algorithm. Compared with other optimization algorithms, the genetic algorithm (GA) has higher global performance and more mature convergence analysis methods [34,35]. Therefore, this paper uses the GA optimization algorithm to search for the optimal parameters of *K* and α in the VMD decomposition process.

The process the genetic algorithm implements to solve the optimization problem is to use the population search technology to treat the population as a set of problem solutions, to generate a new population by applying similar biogenetic operations to the current population, and to gradually make the population contain the approximate optimal solution. The population in the genetic algorithm is the objective function and the fitness function is the function value used for evaluation, so it is especially important to construct a fitness function to be used as the judgment function to generate the optimal solution.

In this paper, the sample entropy (SampEn) is used as the fitness function. Sample entropy is a measure of time series complexity by the magnitude of the probability of generating new patterns in the signal, and the more complex the time series, the larger the calculated value of sample entropy. The expression of sample entropy is as follows:

(7) $$S(m,r)=-\ln\left[\frac{A^{m}(r)}{B^{m}(r)}\right]$$

where *m* is the reconstruction dimension, which usually takes the value of 1, *r* is the threshold size in the range of 0.1σ~0.25σ, σ is the standard deviation, and $A^{m}(r)$, $B^{m}(r)$ are the averages of two signal sequences.

After the signal is decomposed by VMD, the sample entropy of each subsequence is calculated. The sequence with the smallest sample entropy is the trend term of the decomposed sequence. When the decomposition number K is small, the decomposition is insufficient, and other interference terms are mixed into the trend term, resulting in an increase in sample entropy. When the appropriate value of K is taken, the sample entropy of the trend term becomes smaller, so the parameter combination that obtains the minimum sample entropy in the decomposition results is regarded as the best parameter combination in the VMD decomposition process.

### 2.3. Principle of Dual-Channel Extraction of Effective Physiological Information

**Figure 1:**
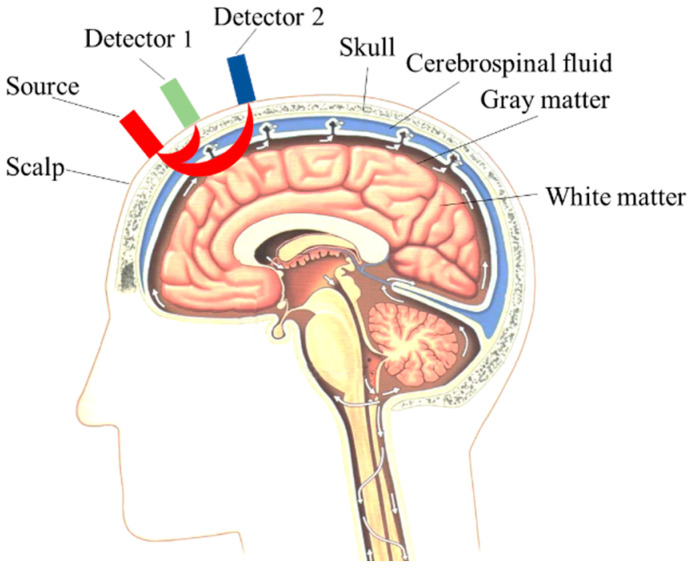
Dual-detector blood oxygen collection mode and head structure model.

Figure 1 shows the schematic diagram of the dual-detector blood oxygen acquisition structure with the head structure model. Generally speaking, the physiological structure of the head mainly consists of five layers. The first layer is the scalp, the second layer is the skull, the third layer is the cerebrospinal fluid, the fourth layer is gray matter, and the fifth layer is white matter. Among these, the scalp and skull are superficial tissues, while gray matter and white matter are deep tissues. The gray matter portion is where human emotions, thoughts, and other cognitive behaviors are generated.

In the following figure, Source is a dual-wavelength light source, Detector 1 (D1) is the short-distance channel detector for measuring the information from the shallow tissue of the head, and Detector 2 (D2) is the long-distance channel detector for detecting the information from the deep tissue.

Since the distance between D1 and the light source is smaller, most of the photons acquired by D1 are photons scattered from the superficial tissue of the scalp, and according to the principle of the dual-detector measurement method, we take the information acquired by D1 as the system interference ($y_{SYS}(t)$). The distance between D2 and the light source is long, and the distance of photon transmission and the depth that can be reached are larger. Therefore, the photons acquired by D2 will carry surface information, deep tissue information, and interference information, and the information from the deep tissue can be used as a component related to the functional activity of the brain ($y_{BFA}(t)$). Therefore, the information measured by the dual channel can be described by the mathematical model of Equations (8) and (9), with the latter being the expanded form of Equation (8):

(8) $$\left[\begin{array}{c} y_{near}(t)\\ y_{far}(t) \end{array}\right]=\left[\begin{array}{cc} k_{1} & 0\\ k_{2} & k_{3} \end{array}\right]\cdot \left[\begin{array}{c} y_{sys}(t)\\ y_{BFA}(t) \end{array}\right]+\left[\begin{array}{c} \varepsilon_{1}(t)\\ \varepsilon_{2}(t) \end{array}\right]$$

(9) $$\left\{\begin{array}{l} y_{near}(t)=k_{1}y_{sys}(t)+\varepsilon_{1}(t)\\ y_{far}(t)=k_{2}y_{sys}(t)+k_{3}y_{BFA}(t)+\varepsilon_{2}(t) \end{array}\right.$$

where $y_{near}(t)$ and $y_{far}(t)$ are the signals measured by detectors D1 and D2, respectively, $k_{1}$, $k_{2}$, and $k_{3}$ are constants, and $\varepsilon_{1}(t)$ and $\varepsilon_{2}(t)$ are the measured noise values of the two channels, respectively.

**Figure 2:**
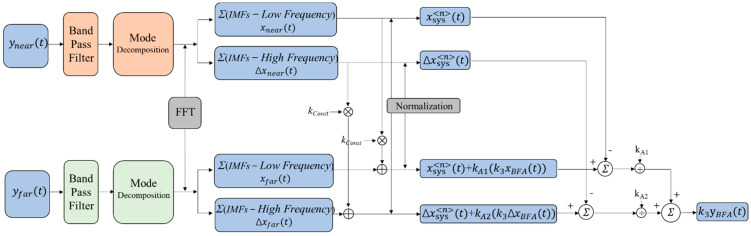
Schematic diagram of the dual-channel algorithm for extracting effective information on brain activity.

Figure 2 is the schematic diagram for extracting effective information about brain activity by using dual detectors. The following are the steps of this method.

Step 1. First, process the acquired signals with band pass filters; then, after the signals are decomposed into various modes, calculate the frequency spectrum of each modal component separately. Finally, combined with the characteristics and spectrum of the actual signal, distinguish the high-frequency and low-frequency components of the two signals.

Step 2. Calculate the sum of the high-frequency component ($\Delta x_{near}(t)$, $\Delta x_{far}(t)$) and low-frequency component ($x_{near}(t)$, $x_{far}(t)$), which are in the short-distance channel and long-distance channel, respectively.

Step 3. First, multiply both $\Delta x_{near}(t)$ and $x_{near}(t)$ by a constant kconst, then add them, respectively, to $\Delta x_{far}(t)$ and $x_{far}(t)$. Second, normalize the results to obtain $\Delta x_{sys}^{<n>}(t)+k_{A2}(k_{3}\Delta x_{BFA}(t))$ and $x_{sys}^{<n>}(t)+k_{A1}(k_{3}x_{BFA}(t))$. Simultaneously, normalize $\Delta x_{near}(t)$ and $x_{near}(t)$ to obtain $\Delta x_{sys}^{<n>}(t)$ and $x_{sys}^{<n>}(t)$.

Step 4. Calculate the results of $x_{sys}^{<n>}(t)+k_{A1}(k_{3}x_{BFA}(t))-x_{sys}^{<n>}(t)$ and $\Delta x_{sys}^{<n>}(t)+k_{A2}(k_{3}\Delta x_{BFA}(t))-\Delta x_{sys}^{<n>}(t)$, and then divide each of these two results by the coefficients K_A1_ and K_A2_, respectively.

Step 5. Add the two results obtained in Step 4 to obtain the final extracted result $k_{3}y_{BFA}$.

(10) $$k_{3}y_{BFA}(t)=k_{3}x_{BFA}(t)+k_{3}\Delta x_{BFA}(t)$$

The detailed derivation of the algorithm is in Appendix A at the end of this paper.

$y_{BFA}(t)$, obtained from Step 5 (Equation (10)), represents the signal of physiological activity in the fourth layer of the brain tissue. In fact, the output of this method is multiply related to the actual cerebral hemodynamic response, so the true value of $k_{3}$ can be obtained by experimental calibration. For the final output result, a filter can be set according to the actual situation to smooth the signal, in order to obtain a clear brain activity information curve.

## 3. Experimental Results and Analysis

### 3.1. Using Simulated Signals to Validate the Proposed Method

#### 3.1.1. Generating the Hemodynamic Response and Physiological Interference

In each layer of brain tissue, if physiological interference caused by the cardiac cycle and respiration is considered, changes in hemodynamic parameters can be simulated by fluctuations *a(t)* caused by the cardiac cycle, fluctuations *b(t)* caused by respiration, and the hemodynamic response *u(t)* of the brain, where the frequencies of *a(t)* and *b(t)* are around 1.2 Hz and 0.25 Hz, respectively [36]. During actual brain function testing, the outer brain tissue carries physiological disturbances that are not associated with the deeper tissue. To simulate such phenomena, a slowly varying random time series τ(t) was added to the scalp layer as its response signal to independent interference. To increase the physiological disturbances caused by low-frequency oscillations and ultra-low-frequency oscillations, *m(t)* and *v(t)* were added to the disturbance components, where *m(t)* denotes low-frequency oscillations with a frequency of 0.1 Hz and *v(t)* denotes ultra-low-frequency oscillations with a frequency of 0.04 Hz. Taking the concentration of oxygenated hemoglobin (Oxy−Hb) as an example, the blood oxygen concentrations in each layer of brain tissue can be expressed as follows [37]:

(11) $$\begin{array}{l} C_{oxy-Hb}^{1}(t)={\left(oxy-Hb\right)}_{base}^{1}+\tau (t)\left[\alpha_{oxy-Hb}^{1}a(t)+\beta_{oxy-Hb}^{1}b(t)+\gamma_{oxy-Hb}^{1}m(t)+\zeta_{oxy-Hb}^{1}v(t)+\varphi_{oxy-Hb}^{1}u(t)\right]\\ C_{oxy-Hb}^{2,3,4,5}(t)={\left(oxy-Hb\right)}_{base}^{2,3,4,5}+\alpha_{oxy-Hb}^{2,3,4,5}a(t)+\beta_{oxy-Hb}^{2,3,4,5}b(t)+\gamma_{oxy-Hb}^{2,3,4,5}m(t)+\zeta_{oxy-Hb}^{2,3,4,5}v(t)+\varphi_{oxy-Hb}^{2,3,4,5}u(t) \end{array}$$

where $C_{Oxy-Hb}^{1}(t)$ and $C_{Oxy-Hb}^{2,3,4,5}(t)$ represent the concentration of Oxy−Hb, and the upper corner marks 1–5 represent the scalp, skull, cerebrospinal fluid, gray matter, and white matter, respectively. ${\left(oxy-Hb\right)}_{base}$ represents the baseline concentration, and the coefficients α,β,γ,ζ,φ are the control parameters of the amplitude of hemodynamic change. Taking the calculation of Oxy−Hb as an example, the parameter values for the physiological activity in each layer are shown in Table 1.

In this paper, simulation signals are used to evaluate the performance of the proposed algorithm. In this simulation, physiological interference ($y_{sys}$) is simulated as the sum of signals with different amplitudes and frequencies representing disturbances related to the heart, breathing, oscillations, and evoked responses. The expression of physiological interference ($y_{sys}$) is shown in Equation (12); $y_{C_{Oxy-Hb}}$ indicates the concentration of oxygenated hemoglobin:

(12) $$y_{sys}=y_{C_{Oxy-Hb}^{1}}+y_{C_{Oxy-Hb}^{2}}+y_{C_{Oxy-Hb}^{3}}$$

Combining Equations (8) and (9), the signals measured by the short-distance channel and long-distance channel detectors can be written as follows:

(13) $$y_{near}=k_{1}(y_{C_{Oxy-Hb}^{1}}+y_{C_{Oxy-Hb}^{2}}+y_{C_{Oxy-Hb}^{3}})+\varepsilon_{1}$$

(14) $$y_{far}=k_{2}(y_{C_{Oxy-Hb}^{1}}+y_{C_{Oxy-Hb}^{2}}+y_{C_{Oxy-Hb}^{3}})+k_{3}y_{C_{Oxy-Hb}^{4}}+\varepsilon_{2}$$

where $\varepsilon_{1}$ and $\varepsilon_{2}$ are two independent noise sources. In $y_{far}$, $y_{C_{Oxy-Hb}^{4}}$ is the effective physiological signal, which is the signal that needs to be extracted eventually. Although $y_{C_{Oxy-Hb}^{1}}$, $y_{C_{Oxy-Hb}^{2}}$, and $y_{C_{Oxy-Hb}^{3}}$ are also physiological signals produced by the human body, they are still regarded as interference signals in the process of extracting effective signals. Therefore, in $y_{far}$, only $y_{C_{Oxy-Hb}^{4}}$ is a valid signal, and the remaining components ($y_{C_{Oxy-Hb}^{1}}+y_{C_{Oxy-Hb}^{2}}+y_{C_{Oxy-Hb}^{3}}+\varepsilon_{2}$) are interference signals. Adjusting the SNR of the signal is achieved by adjusting the ratio between $y_{C_{Oxy-Hb}^{4}}$ and the remaining components.

#### 3.1.2. Compared Methods and Evaluation Index

In order to verify the ability of the proposed method to recover the original signal, the extraction results of the proposed method are now compared with those of RLS, fast-ICA, and EEMD-RLS, and different metrics are used to evaluate the extraction effects of the different methods.

The evaluation metrics used in this paper are the correlation coefficient (R), root-mean-square error (RMSE), and mean absolute error (MAE), and their expressions are shown as follows:

(15) $$R=\frac{\sum_{n=1}^{N}\left[\left(x(n)-\bar{x}\right)\right]\left[\hat{y}(n)-\bar{\hat{y}}\right]}{\sqrt{\sum_{n=1}^{N}{\left[\left(x(n)-\bar{x}\right)\right]}^{2}}\sqrt{\sum_{n=1}^{N}{\left[\hat{y}(n)-\bar{\hat{y}}\right]}^{2}}}$$

where x(n) is the measured signal, $\bar{x}$ is the average of the measured signal, $\hat{y}(n)$ is the extracted signal, $\bar{\hat{y}}$ is the average of the extracted signal, and *N* denotes the length of the signal. The correlation coefficient indicates the similarity of the signals, and if the value of *R* is larger it means that the correlation between the two signals is higher. The expressions of the root-mean-square error and mean absolute error are shown in Equations (16) and (17), where $\hat{y}$ is the predicted value and *y* is the actual value. The smaller the values of the RMSE and MAE, the closer the extracted signal is to the real signal.

(16) $$RMSE=\sqrt{\frac{1}{N}\sum_{n=1}^{N}{\left(\hat{y}-y\right)}^{2}}$$

(17) $$MAE=\frac{1}{N}\sum_{n=1}^{N}\left|\hat{y}-y\right|$$

#### 3.1.3. Simulation Results and Evaluation Analysis

**Figure 3:**
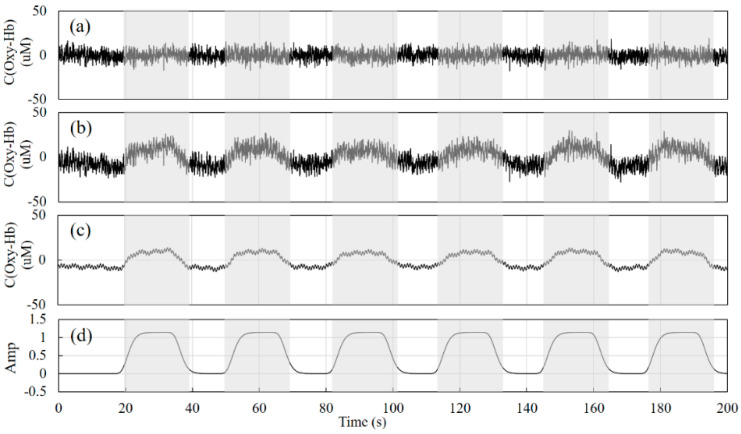
Simulation signals. (**a**) is the simulated signal of the short-distance detector, (**b**) is the simulated signal of the long-distance detector, (**c**) is the physiological activity signal of the gray matter layer, and (**d**) is the real physiological excitation signal.

Taking the signal at SNR = 1 as an example, Figure 3a,b show the signals of the short-distance channel and long-distance channel detectors generated according to Equations (13) and (14), respectively, Figure 3c shows the signal of the physiological activity of the gray matter layer obtained according to Equation (11), and Figure 3d shows the real physiological excitation process. The gray areas in the figure indicate the stimulation phase and the white areas indicate the resting phase. The duration of the test is 200 s, and the duration of each excitation phase is about 20 s.

**Figure 4:**
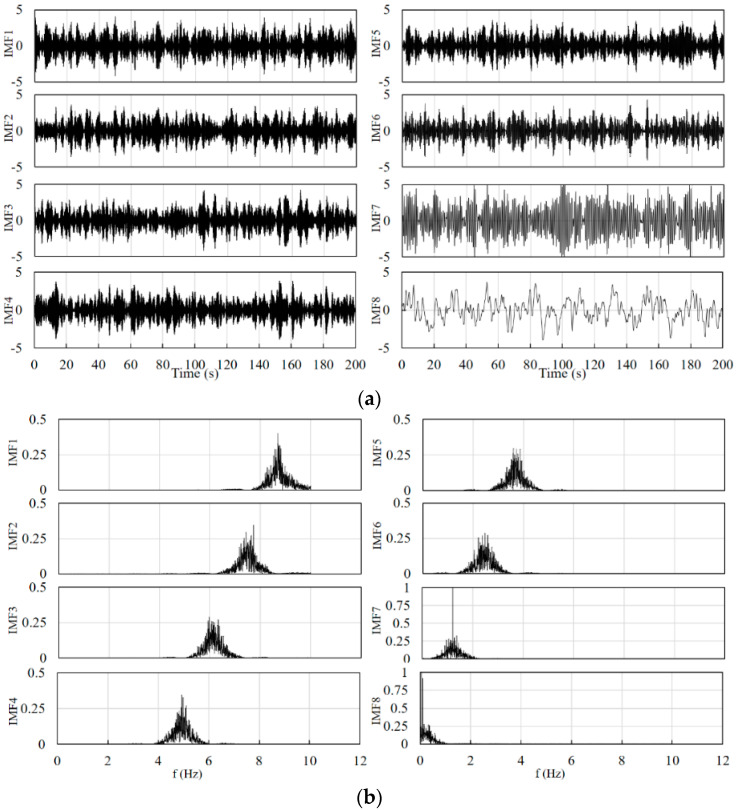
(**a**) Short-distance channel signal decomposition results. (**b**) Spectrum corresponding to the decomposition results of the short-distance channel signal.

**Figure 5:**
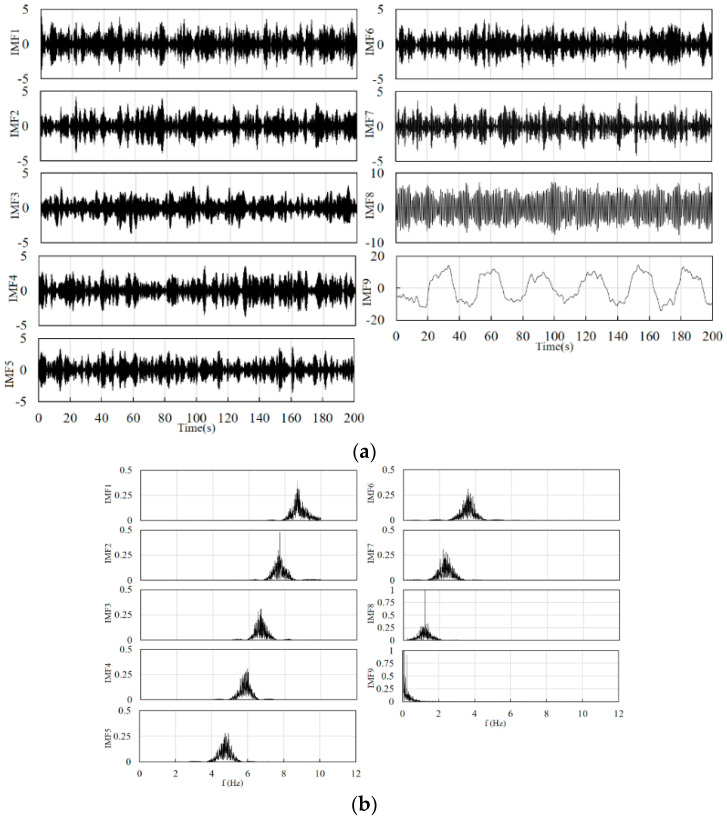
(**a**) Long-distance channel signal decomposition results. (**b**) Spectrum corresponding to the decomposition results of the long-distance channel signal.

Combined with the schematic in Figure 2, the filter used in the front end is a fourth-order Butterworth band pass filter for eliminating instrument noise and baseline drift. The number of signal decompositions should be determined before the VMD decomposition. After testing, the range of the number of decompositions for the cerebral blood oxygen signal can be set as 4–10, and the penalty factor is set between 500 and 2500. The specific decomposition parameters are given by the GA optimization algorithm according to the signal characteristics and the fitness function. The value of the constant *k_const_* is set to 50. The constants *k*_1_, *k*_2_, and *k*_3_ in Equations (13) and (14) are set to 0.8, 0.7, and 0.6, respectively. Figure 4 and Figure 5 show the results of the decomposition of the short-distance channel and long-distance channel signals using VMD and the corresponding spectrograms, respectively. From the spectrum plots in Figure 4 and Figure 5, it can be seen that the VMD can clearly identify each frequency component of the signal after the GA has optimized the decomposition parameters. Combining the long-distance channel and short-distance channel signal characteristics and the corresponding spectra, for the short-distance channel signal, IMF1–IMF7 are selected as the high-frequency components, and IMF8 is the low-frequency component. For the long-distance channel signal, IMF1–IMF8 are selected as the high-frequency components, and IMF9 is the low-frequency component.

**Figure 6:**
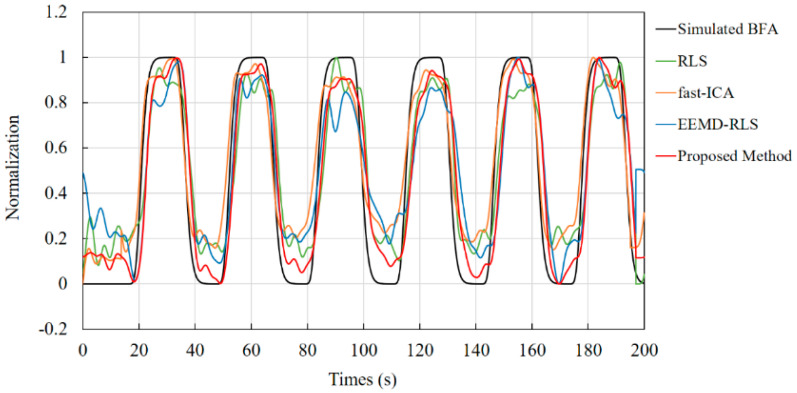
Comparison of results for physiological activity signals and real signals obtained by the proposed method and compared methods.

The results shown in Figure 6 can be obtained by processing the physiological signals (long-distance channel signals and short-distance channel signals) shown in Figure 3. The curves shown in Figure 6 are the normalized comparison results of the effective physiological signals obtained by the proposed method, fast-ICA, RLS, and EEMD-RLS with the simulated generated physiological signals, respectively. To demonstrate the performance of the proposed method, Table 2 compares the test results of the four methods under different signal-to-noise ratio conditions. Combined with Figure 6 and Table 2, it can be found that the method proposed in this paper is superior to the compared methods in waveform shape, amplitude recovery, and various comparison parameters, and can still be used to extract physiological activity signals generated by brain activity to the greatest extent under low SNR conditions.

This paper also investigates the effect of random noise level on the extraction results, the effect of different sensitivity parameters on the extraction results, and the extraction results when the signal-to-noise ratio is less than one. The related test results can be viewed in the Supplementary Materials of this paper.

### 3.2. Using Measured Signals to Validate the Proposed Method

#### 3.2.1. Compared Methods and Evaluation Index

This section will evaluate the performance of the proposed method on real data by extracting information on brain activity from the actual measured data. In this study, fNIRS was used to record information on brain activity in the prefrontal areas of subjects playing a massively multiplayer online competitive combat game under natural conditions. Studies found that the areas of brain activity activation during the game were mainly concentrated in the ventral lateral prefrontal cortex (VLPFC), dorsolateral prefrontal cortex (DLPFC), and frontopolar area (FPA) [38], and that during this process, various game scenarios triggered hemodynamic responses in specific regions of the prefrontal cortex (PFC), which caused Oxy-Hb and Deoxy-Hb concentration changes in the blood.

The whole test lasted about 30 min. In order to distinguish the resting state and the stimulation state, the whole test process was divided into several stages, and the resting and stimulation processes were alternated to highlight the trend of changes in the two hemoglobin concentrations. Before the stimulation test, a resting test period was required, during which there was no stimulation and the subjects needed to remain quiet and calm to enable them to reach a calm state, and then the corresponding stimulation test was conducted. In order to ensure the rigor of the experimental results and eliminate the potential influence of gender differences, we recruited six participants, including three males and three females, with an age range of 24.0 ± 3.0 years. All participants had no neurological or psychiatric conditions and no known history of movement disorders. Before the experiment, the subjects were informed of the relevant test content and process, and agreed to participate in the test research. The experimental process and experimental equipment met safety requirements and could not cause harm to the human body.

**Figure 7:**
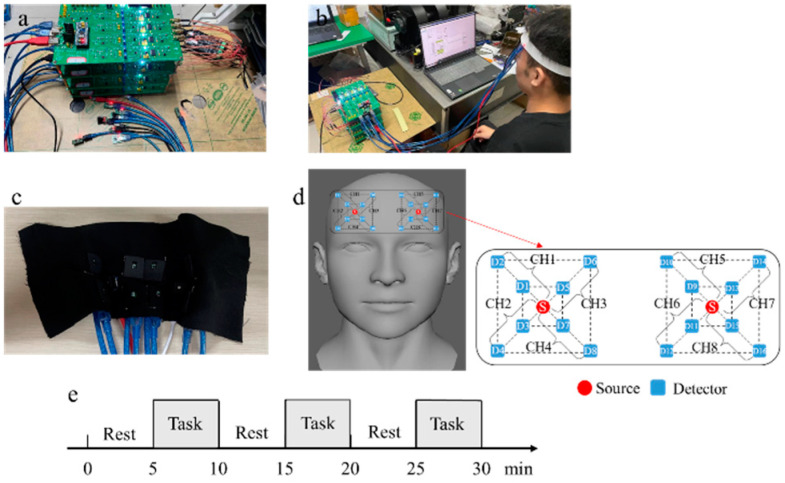
(**a**) Multichannel cerebral oxygen measurement device. (**b**) Photograph taken during testing. (**c**) Position of light source and detector arrangement. (**d**) Arrangement of light sources and detectors for multichannel acquisition devices. (**e**) Block design paradigm.

The multichannel fNIRS measurement and acquisition system used in the experiment is a customized product designed according to the requirements of the experiment. The device has eight measurement channels and two light sources, and each measurement channel contains a long-distance channel and a short-distance channel. The distances between the light source and the detector are 1 cm (short-distance channel) and 3 cm (long-distance channel), respectively. The light source is an integrated dual-wavelength LED light source with wavelengths of 735 nm and 850 nm. The sampling rate of the ADC part is 10 Hz. The system uses time division multiplexing technology to control the light source and separate the signal. The positioning of the light source and detectors was based on the position of Fp2 in the International EEG 10–20 system. During the test, the light source and detector should be fixed on the forehead, and relative movement between the light source, detector, and skin should be avoided during the measurement. Figure 7a shows the multichannel cerebral blood oxygen measurement device, Figure 7b shows a photograph taken during the test, Figure 7c shows the position of the light source and detector arrangement, Figure 7d shows the arrangement of light sources(S) and detectors(D) for multichannel acquisition devices, CHX is the measure channels, and Figure 7e shows the block design paradigm. In Figure 7d, S represents dual-wavelength light sources; odd-numbered detectors are short-distance channel detectors, and even-numbered detectors are long-distance channel detectors. The gray shaded area in Figure 7e represents the stimulation phase of the game and the blank area represents the resting phase.

**Figure 8:**
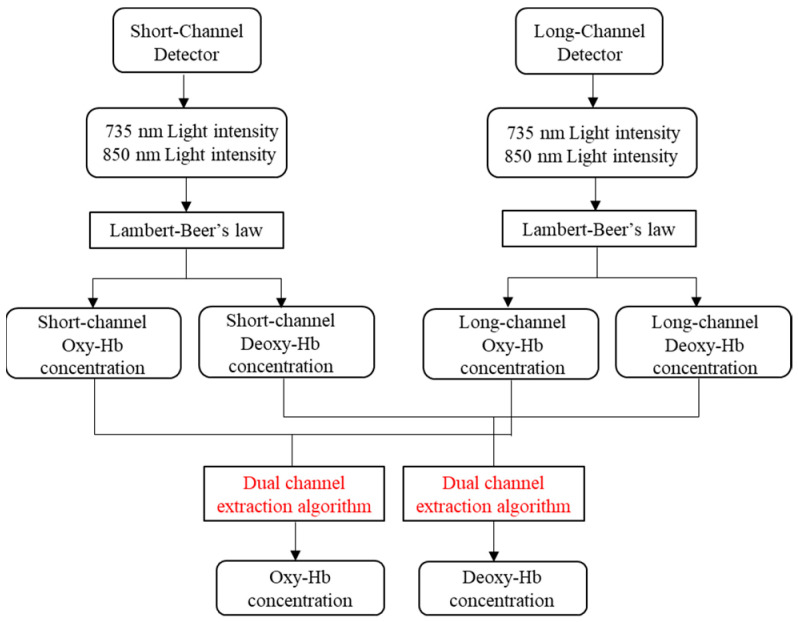
The flowchart of dual-channel fNIRS for extracting effective brain activity information based on cerebral blood oxygenation signals.

Figure 8 shows the flowchart of dual-channel fNIRS for extracting effective brain activity information based on cerebral blood oxygenation signals. First, the signals collected by the detectors are separated to obtain the light intensity signals of the 735 nm and 850 nm light sources. Then, the concentrations of Oxy-Hb and Deoxy-Hb are obtained according to the Lambert–Beer law. Finally, the information extraction algorithm proposed in this paper is utilized to obtain the real brain activity information from the cerebral blood oxygenation signal.

#### 3.2.2. Experimental Results and Analysis

**Figure 9:**
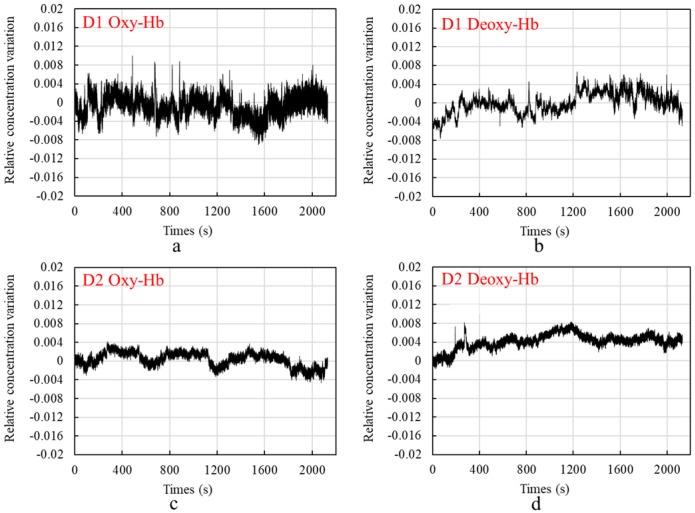
Graph of measurement results: (**a**) shows the change in the relative concentration of Oxy-Hb measured by D1, (**b**) shows the change in the relative concentration of Deoxy-Hb measured by D1, (**c**) shows the change in the relative concentration of Oxy-Hb measured by D2, and (**d**) shows the change in the relative concentration of Deoxy-Hb measured by D2.

Figure 9 shows the graphs of the test results, in which Figure 9a shows the change in the relative concentration of Oxy-Hb measured by D1, Figure 9b shows the change in the relative concentration of Deoxy-Hb measured by D1, Figure 9c shows the change in the relative concentration of Oxy-Hb measured by D2, and Figure 9d shows the change in the relative concentration of Deoxy-Hb measured by D2.

**Figure 10:**
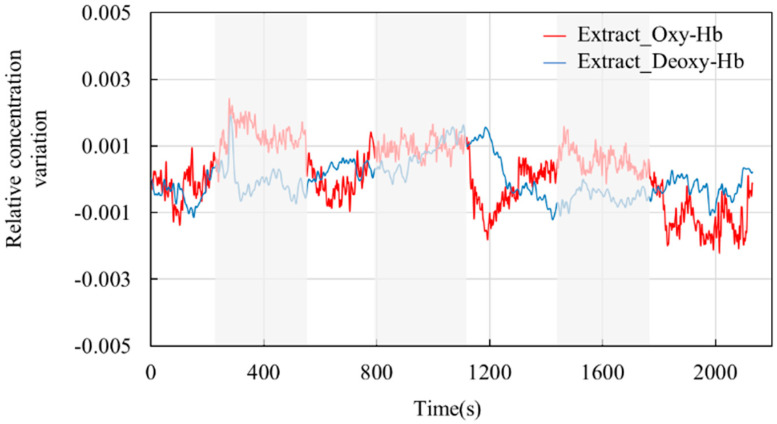
Extraction results. The red line shows the results for the change in the relative concentration of Oxy-Hb. The blue line shows the results for the change in the relative concentration of Deoxy-Hb.

**Figure 11:**
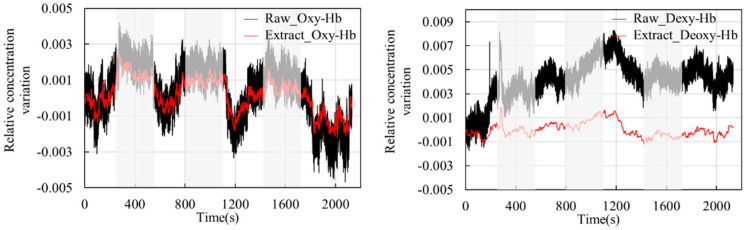
Comparison between the raw signal and the extracted result.

Figure 10 shows the blood oxygen signal obtained by the dual-channel effective information extraction method, where the red line is the extraction result for Oxy-Hb, and the blue line is the extraction result for Deoxy-Hb. Figure 11 shows the comparison between the original signal and the extracted result. The shaded part represents the time period during which the task stimulation was performed during the actual measurement, and the rest represents the time period of the resting state.

From the extraction results in Figure 10 and Figure 11, it can be observed that over the entire time series, the concentrations of the two types of hemoglobin exhibit a roughly symmetric trend, and the durations corresponding to each state are generally consistent with the planned durations of each stage. From the graphs, it can be seen that during the initial resting phase, the concentrations of both oxyhemoglobin (Oxy-Hb) and deoxyhemoglobin (Deoxy-Hb) remain stable. When the first stimulation task begins, there is a rapid increase in Oxy-Hb concentration, which then stabilizes at a high level. The duration of this high-level state corresponds approximately to the duration of the stimulation phase. Upon the cessation of stimulation and entry into the resting phase, Oxy-Hb concentration gradually returns to a lower level. During this process, the changes in Deoxy-Hb concentration exhibit an opposite trend to those in Oxy-Hb, consistent with the analysis based on the neurovascular coupling mechanism. However, the change in Deoxy-Hb concentration during this process is smaller than that in Oxy-Hb concentration. From a brain activity perspective, when stimulation occurs, the brain responds to the corresponding stimuli, leading to increased oxygen demand in brain tissue, resulting in increased arterial blood flow and subsequently increased Oxy-Hb concentration. From a physiological interference perspective, the smaller change in Deoxy-Hb concentration suggests that physiological interference has a relatively lower impact on Deoxy-Hb concentration. This could be explained by physiological interference mainly originating from arterial blood, which has a higher proportion of Oxy-Hb compared with venous blood, thereby introducing a greater degree of physiological interference in the measurement results for Oxy-Hb.

**Figure 12:**
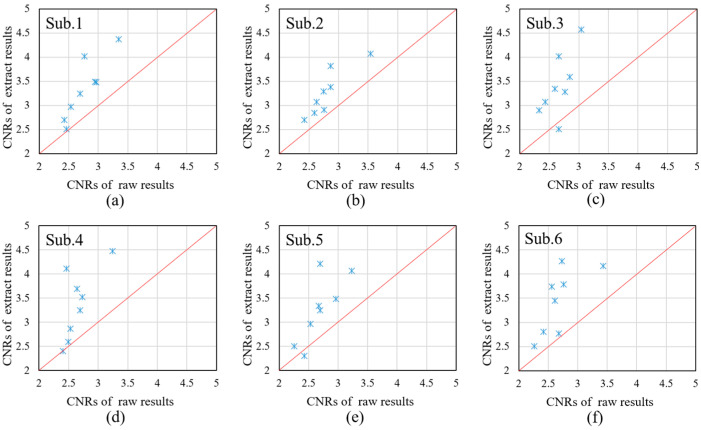
Comparison of CNRs between raw Oxy-Hb and the Oxy-Hb estimated using the extraction results. Sub.1-Sub.6 indicates the test results for the six subjects. (**a**–**c**): male subjects, (**d**–**f**): female subjects.

For the measured signal, the real signal cannot be known, so the evaluation index mentioned in the previous section can no longer be used to evaluate the signal quality. Here, we used the CNR (Contrast-to-Noise Ratio) to evaluate the quality of the extracted signals [39]. A high CNR value indicates a high ratio of signal to noise in the task. The CNR is calculated as follows:

(18) $$CNR=\frac{mean(task)-mean(rest)}{\sqrt{\mathrm{var}(task)+\mathrm{var}(rest)}}$$

where “task” represents the task period and “rest” represents the rest time. We intercepted the blood oxygen concentrations in the task state and the rest state in each channel, and calculated the CNR values of each channel, respectively. Taking the calculation of Oxy-Hb as an example, the results are shown in Figure 12. Figure 12a–f show the test results for the six subjects, respectively; Sub.1–Sub.3 are the male subjects, and Sub.4–Sub.6 are the female subjects. The horizontal coordinate represents the CNR value of the long-distance channel, and the vertical coordinate represents the CNR value of the extraction result.

Figure 12 compares the CNRs of the raw Oxy-Hb and the Oxy-Hb obtained by the proposed method across six subjects. By comparing the CNRs of the original signal with the CNRs of the extracted result, it can be found that the method proposed in this paper can effectively suppress the interference components in the original signal and extract the active components of the cerebral blood oxygen signal to the maximum extent. The Supplementary Materials provided with this paper offer a performance comparison of different extraction methods under actual measurement conditions.

## 4. Discussion

Physiological interference can greatly reduce the performance of fNIRS in measuring evoked brain activity responses. There are many ways to solve this problem. The common method is to identify and separate the interference components in the fNIRS signal, and use the filtering algorithm to eliminate the interference. The disadvantage of this method is that it requires more additional equipment. Another approach is adaptive filtering, which uses existing reference signals to separate brain activity responses from physiological disturbances. However, due to individual differences, this approach is not appropriate. Therefore, this paper designs a dual-channel effective information extraction algorithm based on multi-distance measurement to eliminate physiological interference in the measurement results. The advantage of this method is that it can be used to effectively extract real physiological activity signals in real time.

It should be noted that the purpose of using the proposed algorithm for signal extraction is the modal decomposition of the signal, and the quality of the decomposition results will directly affect the quality of the extraction results. The purpose of using VMD to decompose the signal in this paper is to ensure the singularity of the components of each decomposition result and to avoid modal aliasing in the decomposition results. In addition, the reference signal of the algorithm comes from the subject; therefore, the extraction results are not affected by the subject’s physical condition or physiological state, thus improving the accuracy of the results and the robustness of the system.

When extracting brain activity signals, the extraction results can not only detect the brain’s functional signals, but also analyze the characteristics of the brain’s outer physiological signals through mode decomposition. By rationally arranging the detector distribution, it is also possible to obtain maps of brain activity corresponding to different physiological states.

## 5. Conclusions

This paper designs a new extraction method based on the dual-channel method and compares various extraction methods using synthetic physiological signals. The results show that the proposed method can effectively reduce various physiological interferences in the measured signals and can still obtain a good extraction effect even under low SNR conditions. In this paper, we also designed a relevant brain activity stimulation experiment to validate the proposed method using the measured data, and the experimental results show that the method can be used to extract real brain activity information under practical conditions. The proposed method for extracting effective information on brain activity lays the foundation for subsequent research on brain function analysis and brain–computer interface technology.

## Figures and Tables

**Table 1:** Values of physiological activity parameters for each tissue layer.

Tissue	α(μM) Cardiac Cycle	β(μM) Breath	γ(μM) Low-Frequency Oscillation	ζ(μM) Ultra-Low-Frequency Oscillation	φ(μM) Evoked Response
Scalp	0.2	0.6	0.9	1.0	0
Skull	0.2	0.63	0.96	1.1	0
Cerebrospinal fluid	0.02	0.06	0.08	0.1	0
Gray matter	0.2	0.65	0.92	1.1	15
White matter	0.2	0.6	0.9	1.0	0

**Table 2:** Comparison of the performance test results of the four extraction methods.

SNR	Method	Parameter		
		R	rMSE	MAE
SNR = 1	**Proposed method**	**0.98482**	**0.09646**	**0.08337**
	RLS	0.92799	0.18884	0.16952
	fast-ICA	0.98299	0.15231	0.12925
	EEMD-RLS	0.89344	0.22046	0.19163
SNR = 2	**Proposed method**	**0.98567**	**0.09343**	**0.07951**
	RLS	0.91562	0.18987	0.15979
	fast-ICA	0.98508	0.14484	0.12397
	EEMD-RLS	0.96321	0.14814	0.12528
SNR = 4	**Proposed method**	**0.98764**	**0.08761**	**0.07661**
	RLS fast-ICA EEMD-RLS	0.98166 0.97678 0.95345	0.11452 0.17078 0.16344	0.10194 0.14357 0.13802

## Data Availability

The code and data will be made available on request.

## Supplementary Materials

The following supporting information can be downloaded at: https://www.mdpi.com/article/10.3390/s24061820/s1.

## Appendix A. The Derivation Process of the Proposed Method

The following is the derivation process of the proposed method.

The information measured by the dual channel can be described by the mathematical model of Equations (1) and (2):

(A1) $$\left[\begin{array}{c} y_{near}(t)\\ y_{far}(t) \end{array}\right]=\left[\begin{array}{cc} k_{1} & 0\\ k_{2} & k_{3} \end{array}\right]\cdot \left[\begin{array}{c} y_{sys}(t)\\ y_{BFA}(t) \end{array}\right]+\left[\begin{array}{c} \varepsilon_{1}(t)\\ \varepsilon_{2}(t) \end{array}\right]$$

(A2) $$\left\{\begin{array}{l} y_{near}(t)=k_{1}y_{sys}(t)+\varepsilon_{1}(t)\\ y_{far}(t)=k_{2}y_{sys}(t)+k_{3}y_{BFA}(t)+\varepsilon_{2}(t) \end{array}\right.$$

Step 1 is executed for the signal acquired by the short-distance channel detector; i.e., the short-distance channel signal ($y_{near}(t)$) is decomposed into low-frequency components ($x_{near}(t)$) and high-frequency components ($\Delta x_{near}(t)$) by using modal decomposition, and the process can be illustrated by Equation (3):

(A3) $$y_{near}(t)=x_{near}(t)+\Delta x_{near}(t)$$

Combining this with Equation (A1), we can obtain the following:

(A4) $$\left\{\begin{array}{l} y_{near}(t)=x_{near}(t)+\Delta x_{near}(t)\\ y_{near}(t)=k_{1}y_{sys}(t)=k_{1}[x_{sys}(t)+\Delta x_{sys}(t)] \end{array}\right.\Rightarrow \left\{\begin{array}{l} x_{near}(t)=k_{1}x_{sys}(t)\\ \Delta x_{near}(t)=k_{1}\Delta x_{sys}(t) \end{array}\right.$$

For the normalization of the sum of the low-frequency component of the short-distance channel signal, the normalized result of $x_{near}(t)$ is expressed by $x_{near}^{<N>}(t)$, and the normalized result of $\Delta x_{near}(t)$ is expressed by $\Delta x_{near}^{<N>}(t)$. Combined with the result of Equation (4), we can obtain the following:

(A5) $$\begin{array}{ll} x_{near}^{<N>}(t) & =\frac{x_{near}(t)-\min[x_{near}(t)]}{\max[x_{near}(t)]-\min[x_{near}(t)]}\\ & =\frac{k_{1}x_{sys}(t)-\min[k_{1}x_{sys}(t)]}{\max[k_{1}x_{sys}(t)]-\min[k_{1}x_{sys}(t)]}\\ & =\frac{k_{1}x_{sys}(t)-k_{1}\min[x_{sys}(t)]}{k_{1}\max[x_{sys}(t)]-k_{1}\min[x_{sys}(t)]}\\ & =\frac{x_{sys}(t)-\min[x_{sys}(t)]}{\max[x_{sys}(t)]-\min[x_{sys}(t)]}\\ & =x_{sys}^{<N>}(t) \end{array}$$

Similarly, the normalization of the sum of the high-frequency components in the short-distance channel signal can be obtained:

(A6) $$\Delta x_{near}^{<N>}(t)=\Delta x_{sys}^{<N>}(t)$$

where $x_{sys}^{<N>}(t)$ and $\Delta x_{sys}^{<N>}$ are the normalized results of $x_{sys}(t)$ and $\Delta x_{sys}(t)$, respectively.

Step 1 is executed for the signal acquired by the long-distance channel detector; i.e., the long-distance channel signal ($y_{far}(t)$) is decomposed into low-frequency components ($x_{far}(t)$) and high-frequency components ($\Delta x_{far}(t)$) by using modal decomposition, and the process can be illustrated by Equation (7):

(A7) $$y_{far}(t)=x_{far}(t)+\Delta x_{far}(t)$$

Combining this with Equation (A2), we can obtain the following:

(A8) $$\begin{array}{ll} y_{far}(t) & =k_{2}y_{sys}(t)+k_{3}y_{BFA}(t)\\ & =k_{2}[x_{sys}(t)+\Delta x_{sys}(t)]+k_{3}[x_{BFA}(t)+\Delta x_{BFA}(t)]\\ & =k_{2}x_{sys}(t)+k_{3}x_{BFA}(t)+k_{2}\Delta x_{sys}(t)+k_{3}\Delta x_{BFA}(t) \end{array}$$

Combining Equations (7) and (8), we can obtain the following:

(A9) $$\left\{\begin{array}{l} x_{far}(t)=k_{2}x_{sys}(t)+k_{3}x_{BFA}(t)\\ \Delta x_{far}(t)=k_{2}\Delta x_{sys}(t)+k_{3}\Delta x_{BFA}(t) \end{array}\right.$$

The decomposition results $x_{near}(t)$ and $\Delta x_{near}(t)$ of the short-distance channel signal $y_{near}(t)$ are multiplied by the constant *k_const_* and added to the decomposition results $x_{far}(t)$ and $\Delta x_{far}(t)$ of the long-distance channel signal $y_{far}(t)$, respectively, to obtain Equations (10) and (11).

(A10) $$\begin{array}{ll} x_{f}(t) & =x_{far}(t)+K_{const}x_{near}(t)\\ & =k_{2}x_{sys}(t)+k_{3}x_{BFA}(t)+K_{const}[k_{1}x_{sys}(t)]\\ & =K_{G}x_{sys}(t)+k_{3}x_{BFA}(t) \end{array}$$

(A11) $$\begin{array}{ll} \Delta x_{f}(t) & =\Delta x_{far}(t)+K_{const}\Delta x_{near}(t)\\ & =k_{2}\Delta x_{sys}(t)+k_{3}\Delta x_{BFA}(t)+K_{const}[k_{1}\Delta x_{sys}(t)]\\ & =K_{G}\Delta x_{sys}(t)+k_{3}\Delta x_{BFA}(t) \end{array}$$

where $K_{G}=k_{2}+K_{const}\cdot k_{1}$, and $K_{G}>>\;k_{3}$.

Supposing x>>y, we can then consider that max(x+y)≈max(x) and min(x+y)≈min(x). Combining the conclusions, the normalization of x+y can be written as follows:

(A12) $$\begin{array}{ll} {\left(x+y\right)}^{<N>} & =\frac{\left(x+y)-\min(x+y\right)}{\max(x+y)-\min(x+y)}\\ & \approx \frac{\left(x+y)-\min(x\right)}{\max(x)-\min(x)}\\ & =\frac{x-\min(x)}{\max(x)-\min(x)}+\frac{y}{\max(x)-\min(x)} \end{array}$$

If $K_{A}=\frac{1}{\max(x)-\min(x)}$, then the normalized result of x+y can be written as follows:

(A13) $${\left(x+y\right)}^{<N>}=x^{<N>}+K_{A}\cdot y$$

According to the conclusion of Equation (13), the normalized results of $x_{f}(t)$ (Equation (10)) and $\Delta x_{f}(t)$ (Equation (11)) can be written as follows:

(A14) $$\begin{array}{ll} x_{f}^{<N>}(t) & ={\left[K_{G}x_{sys}(t)+k_{3}x_{BFA}(t)\right]}^{<N>}\\ & ={\left[K_{G}x_{sys}(t)\right]}^{<N>}+K_{A1}[k_{3}x_{BFA}(t)]\\ & \approx x_{sys}^{<N>}(t)+k_{A1}k_{3}x_{BFA}(t) \end{array}$$

(A15) $$\begin{array}{ll} \Delta x_{f}^{<N>}(t) & ={\left[K_{G}\Delta x_{sys}(t)+k_{3}\Delta x_{BFA}(t)\right]}^{<N>}\\ & ={\left[K_{G}\Delta x_{sys}(t)\right]}^{<N>}+K_{A2}[k_{3}\Delta x_{BFA}(t)]\\ & \approx \Delta x_{sys}^{<N>}(t)+k_{A2}k_{3}\Delta x_{BFA}(t) \end{array}$$

The expressions of $K_{A1}$ and $K_{A2}$ are as follows:

(A16) $$\left\{\begin{array}{l} K_{A1}=\frac{1}{\max[K_{G}x_{sys}(t)]-\min[K_{G}x_{sys}(t)]}\\ K_{A2}=\frac{1}{\max[K_{G}\Delta x_{sys}(t)]-\min[K_{G}\Delta x_{sys}(t)]} \end{array}\right.$$

The result of the normalization of the long-distance channel signal is subtracted from the result of the normalization of the short-distance channel signal, as shown in Equations (A5), (A6), (A14), and (A15).

(A17) $$x_{f}^{<N>}(t)-x_{near}^{<N>}(t)=x_{sys}^{<N>}(t)+K_{A1}[k_{3}x_{BFA}(t)]-x_{sys}^{<N>}(t)=K_{A1}[k_{3}x_{BFA}(t)]$$

(A18) $$\Delta x_{f}^{<N>}(t)-\Delta x_{near}^{<N>}(t)=\Delta x_{sys}^{<N>}(t)+K_{A2}[k_{3}\Delta x_{BFA}(t)]-\Delta x_{sys}^{<N>}(t)=K_{A2}[k_{3}\Delta x_{BFA}(t)]$$

Equations (A17) and (A18) are divided by their respective coefficients $K_{A1}$ and $K_{A2}$ and then added together, which allows information to be effectively extracted.

(A19) $$k_{3}y_{BFA}(t)=k_{3}x_{BFA}(t)+k_{3}\Delta x_{BFA}(t)$$

It should be noted that the four constants *k*_1_, *k*_2_, *k*_3_, and *k_const_* involved in the algorithm are only used for calculation, and do not represent the real values of a certain component. Their values can be arbitrarily selected without affecting the final extraction result.
