# Supplementary material for: Effective Signal Extraction Algorithm for Cerebral Blood Oxygen Based on Dual Detectors
Source: Sensors (Basel). 2024 Mar 12;24(6):1820. doi: 10.3390/s24061820 (PMC10974257; doi:10.3390/s24061820)
Supplement: Supplementary file 1 [file sensors-24-01820-s001.zip › sensors-2904413-supplementary.pdf]

1. Supplementary experiments

1.1 Investigating the effect of random noise on extraction results

In this section, 4 groups of control experiments were added while keeping the fourth layer of φ (Evoked response) values constant. The magnitude of random noise(E) is different in each control group. The test also investigates the extraction results for signals with different SNR when different noise amplitudes are chosen. Figure 1 takes the SNR=4 as an example to draw the waveform of the long distance measurement channel with different noise levels. The black line in the figure 2 shows the real physiological signal of the fourth layer. The extraction effects of effective signals with different SNR are shown as red, blue and orange lines. In this study, the value of physiological stimuli φ(Evoked response) in the fourth layer is set to 15.

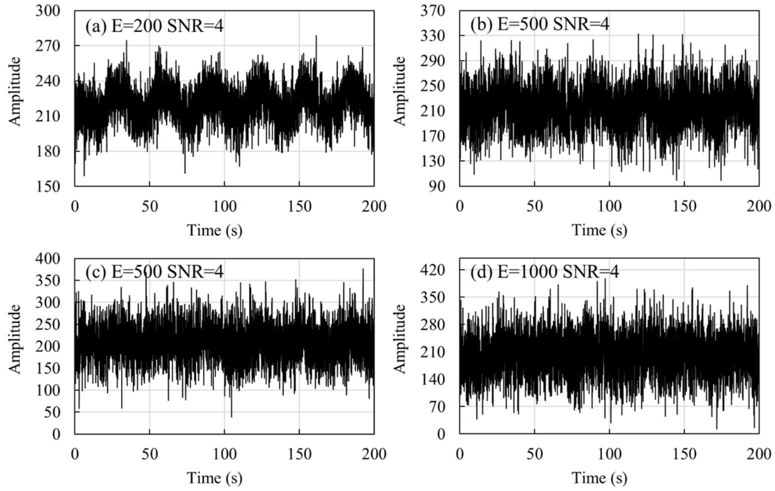

Figure S1. Waveform of long distance measurement channel with different noise levels

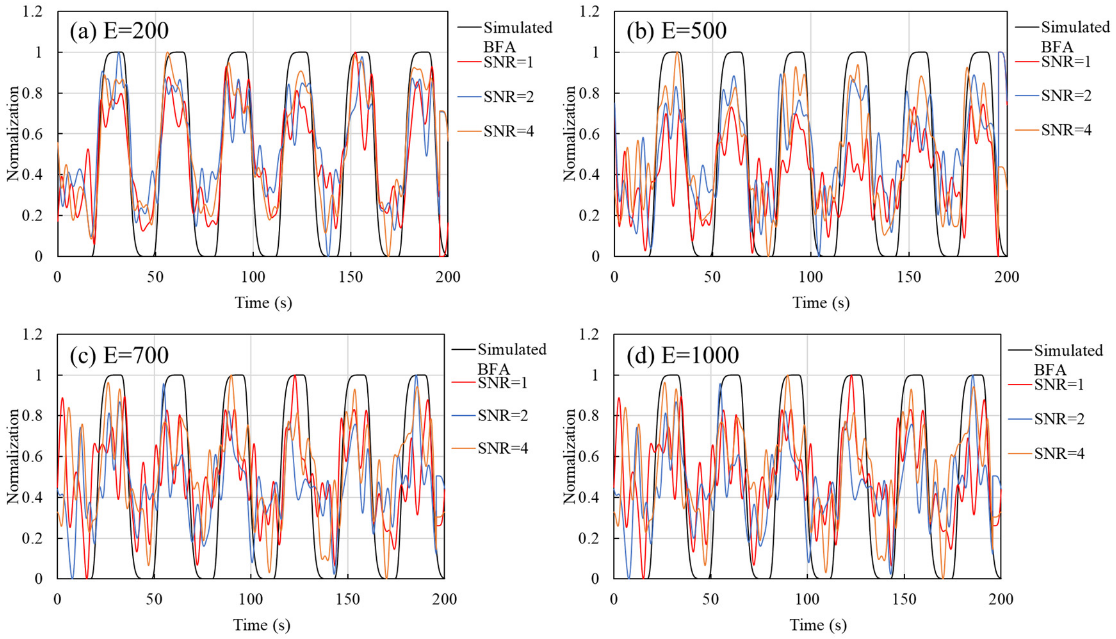

Figure S2. The extraction results of effective signals under different random noise conditions

It can be seen from Figure 2 that with the increase of noise amplitude, the signal extracted by the method proposed in this paper is basically consistent with the excitation process (black line) in terms of variation trend, but the distortion degree of signal details is gradually increasing.

Table S1. Comparison of extraction results under different test conditions

	E=200			E=500			E=700			E=1000		
	SNR =1	SNR =2	SNR =4	SNR =1	SNR =2	SNR =4	SNR =1	SNR =2	SNR =4	SNR =1	SNR =2	SNR =4
R	0.885	0.895	0.926	0.772	0.805	0.872	0.627	0.670	0.788	0.513	0.583	0.734
RMSE	0.261	0.242	0.219	0.302	0.281	0.269	0.352	0.337	0.297	0.384	0.363	0.317
MAE	0.231	0.216	0.183	0.260	0.251	0.240	0.317	0.295	0.258	0.319	0.363	0.271

1.2 Investigating the effect of sensitive parameters on extraction results

The parameters α 、 β、 γ、 ζ in Table 1 of the original paper, can be considered as constant, less affected by physiological changes, and taking close values in the each tissue layers. For the parameter φ it is more affected by physiological activities, therefore, this part investigates the extraction effect of the proposed method when φ takes different values. This part generates signals with different SNR by setting different φ values. The proposed method is used to extract these signals, and the extraction results are evaluated. It should be noted that the amplitude of the random noise is constant during this test. The random noise takes the value of 50. Figure 3 shows the waveform of the long distance measurement channel with different physiological stimulus intensities. Figure 4 shows the extraction results for signals with different characteristics.

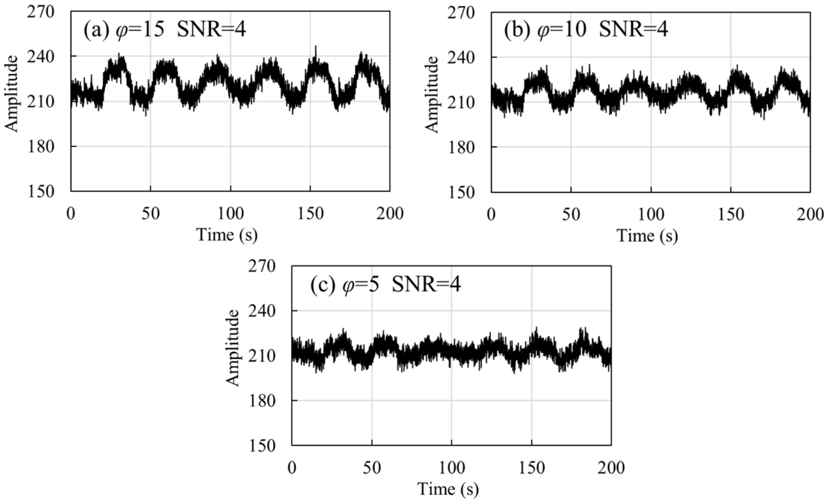

Figure S3. Waveforms of long distance measurement channels with different physiological stimulus intensities

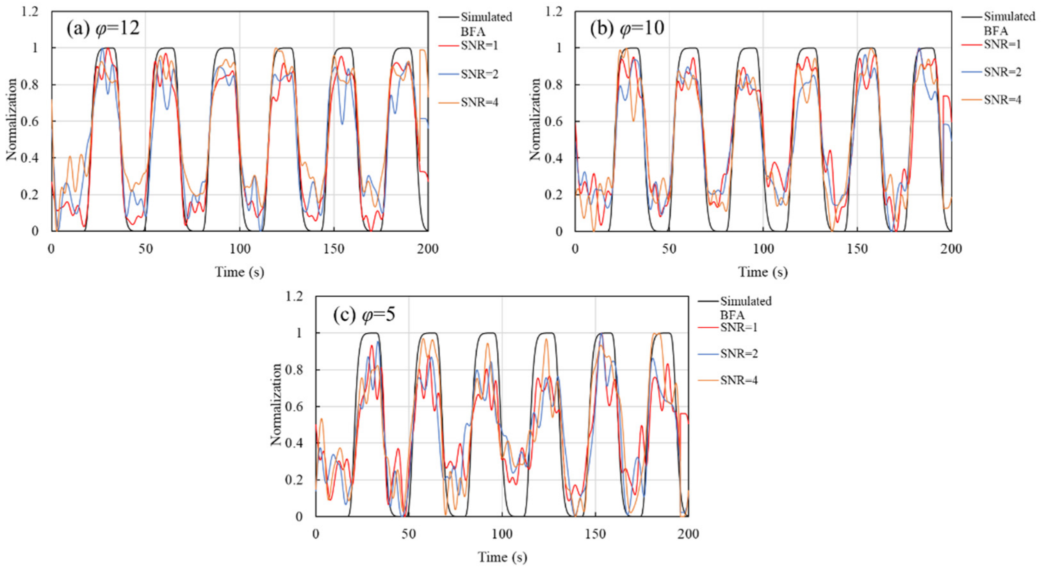

Figure S4. The extraction results of effective signals under different φ value (Evoked response) conditions

It can be seen from figure 4 that with the decrease of physiological stimulus intensity and SNR, the quality of the extracted signal also gradually decreases.

Table S2. Comparison of extraction results under different test conditions

	φ=12			φ=10			φ=5		
	SNR=1	SNR=2	SNR=4	SNR=1	SNR=2	SNR=4	SNR=1	SNR=2	SNR=4
R	0.929	0.950	0.984	0.915	0.936	0.968	0.884	0.890	0.896
RMSE	0.200	0.192	0.116	0.225	0.190	0.158	0.239	0.252	0.215
MAE	0.159	0.166	0.099	0.171	0.158	0.136	0.204	0.224	0.178

1.3 Investigating the extraction effect of the proposed method when the SNR of the signal is <1

This section investigates the extraction effectiveness of the proposed method when the SNR of the signal is less than 1. The test procedure treats the amplitude of the random noise and the physiological stimulus of the fourth layer as constant values. Signals with different SNR are generated by adjusting the ratio of the fourth layer signal to the interference signal. Finally, the proposed method is utilized to extract the effective signal and the extraction results are evaluated by different metrics. During this test, the value of physiological stimuli is set to 15, and the value of random noise is set to 500, 200. Figure 5 shows the waveform of long distance measurement channel when SNR<1. Figure 6 shows the extraction results of the signal with SNR <1 when random noise (E) takes different values.

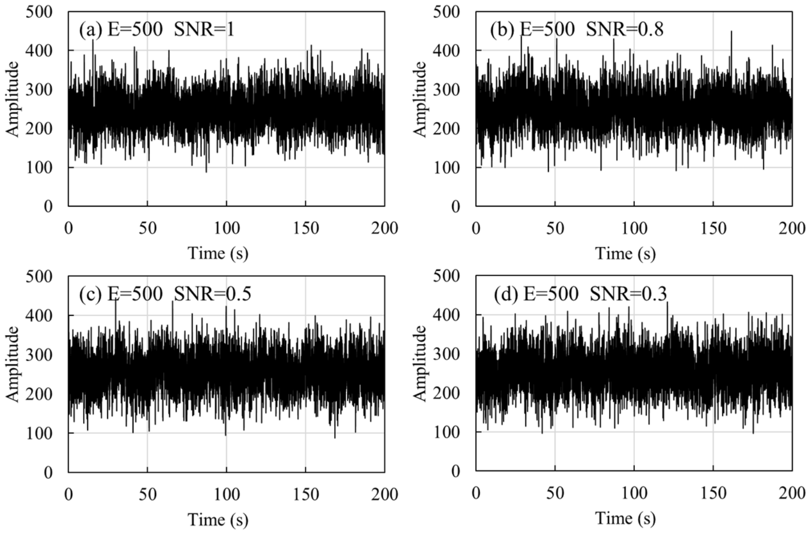

Figure S5. Waveform of long distance measurement channel when SNR<1

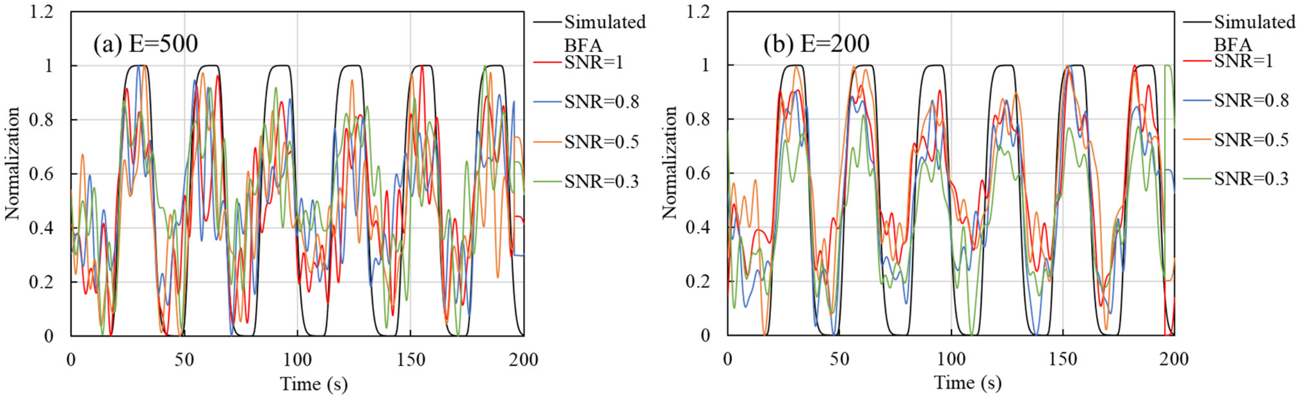

Figure S6. Effective signal extraction results under different SNR conditions

It can be seen from Figure 6 that even in the case of SNR<1, the results extracted by the method proposed in this paper still have a high similarity to the real excitation process in terms of change trend.

Table S3. Comparison of extraction results under different test conditions

	E=200				E=500			
	SNR=1	SNR=0.8	SNR=0.5	SNR=0.3	SNR=1	SNR=0.8	SNR=0.5	SNR=0.3
R	0.903	0.898	0.874	0.784	0.812	0.716	0.713	0.708
RMSE	0.258	0.230	0.274	0.301	0.277	0.323	0.318	0.328
MAE	0.225	0.199	0.229	0.261	0.235	0.279	0.267	0.285

2. Comparison of extraction effect with different extraction methods

In this part, different extraction methods (fast-ICA, EEMD-RLS, RLS) are used to extract the measured signal, and the extraction effect is evaluated by CNR index.

In the figures, Sub.1-Sub.6 indicates 6 subjects, and the blue asterisk indicates the channel of measurement. The larger the value of the vertical coordinate of the blue asterisk in the figures, the better the extraction effect of the signal. On the whole, the extraction effect of fast-ICA is better than the other two methods.

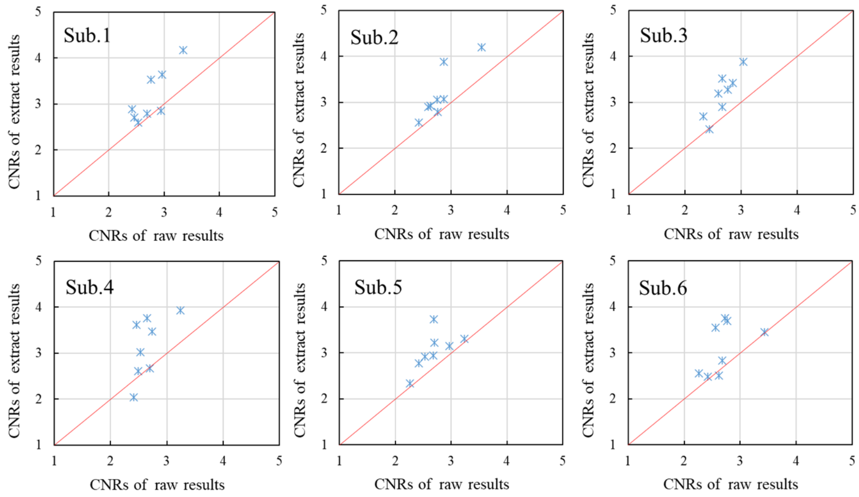

Comparison of CNRs between raw Oxy-Hb and the Oxy-Hb estimated by the extract results with fast-ICA

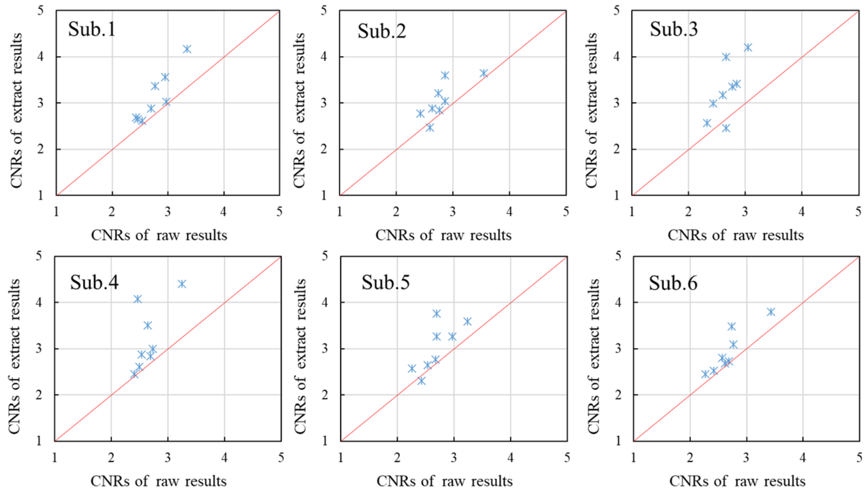

Comparison of CNRs between raw Oxy-Hb and the Oxy-Hb estimated by the extract results with EEMD-RLS

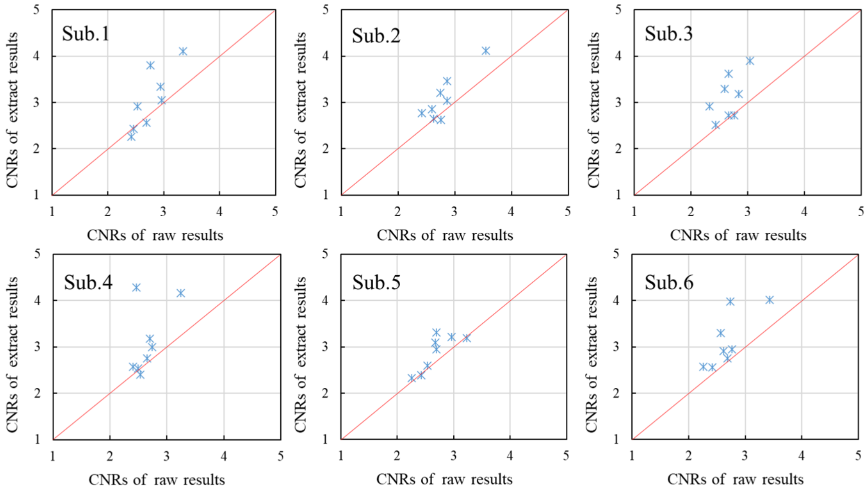

Comparison of CNRs between raw Oxy-Hb and the Oxy-Hb estimated by the extract results with RLS
